# Meta-Analysis and Meta-Regression Indicate Dynamic Prevalence and Moderators of Foodborne Pathogens in African Indigenous Fermented Milk

**DOI:** 10.3390/microorganisms7110563

**Published:** 2019-11-14

**Authors:** Joseph Wambui, Patrick Murigu Kamau Njage, Taurai Tasara, Elna Maria Buys

**Affiliations:** 1 Institute for Food Safety and Hygiene, Vetsuisse Faculty, University of Zurich, Winterthurerstrasse 272, 8057 Zurich, Switzerland; tasarat@fsafety.uzh.ch; 2 Division for Epidemiology and Microbial Genomics, National Food Institute, Technical University of Denmark, Kemitorvet, Building 204, 2800 Kongens Lyngby, Denmark; panj@food.dtu.dk; 3 Department of Consumer and Food Sciences, University of Pretoria, Lynwood Road, Pretoria 0002, South Africa

**Keywords:** food safety, milk fermentation, *Staphylococcus aureus*, *Escherichia coli*, Listeria monocytogenes, *Salmonella* spp.

## Abstract

As more microbiological data for indigenous fermented milk (IFM) becomes available, concern about their microbial safety becomes eminent. Nonetheless, these data are highly fragmented, and a tool is required to integrate existing data and to provide a basis for data-driven decision making for IFM’s safety. Therefore, meta-analysis and meta-regression were conducted to estimate the prevalence of foodborne pathogens in IFM and to determine factors influencing the estimated values. Using Africa as a case, searches were systematically made for published data and relevant grey literature. Data from 18 studies in 15 countries were analyzed. *Staphylococcus aureus* (37%), pathogenic *Escherichia coli* (16%), *Listeria monocytogenes* (6%), and *Salmonella* spp. (3%) were the most prevalent pathogens with a pooled prevalence estimate of 12%. Heterogeneity among prevalence estimates was attributed to sampling point and microbial group but could be moderated by publication year, country cluster, and methods for microbial confirmation. The pooled prevalence estimates increased over time as more studies became available, whereby the odds were higher in studies from 2010 onwards than studies before 2010. From the analyses, *S. aureus* presented the greatest safety concern in African IFM. Future microbiological studies should take into consideration different IFM sampling points and advanced analytical methods to identify pathogens.

## 1. Introduction

Food fermentation is one of the oldest food preservation methods. It has been utilized over the years by different cultures all over the world and continues to be an important source of nutrition among communities. Among the fermented foods, indigenous fermented milk (IFM) plays an important role in the daily diet of communities whose lives are structured around livestock [1]. Besides this, IFM is believed to aid in the control of some diseases among these communities [2]. In particular, one study showed that *Kule naoto* and *Kwerionik*, which are IFMs from Kenya and Uganda, respectively, may have probiotic potential [3]. Another study showed that a diet containing *Maas*, a south African IFM, can confer health benefits among children and old patients [4].

Besides the potential benefits of IFM, their microbial safety is rarely considered because it is assumed that their low pH would naturally control bacterial pathogens [5]. This may not always be the case since foodborne pathogens such as *Listeria monocytogenes* are known to be acid-resistant and, hence, can survive from this and other intrinsic factors within foods [6]. Furthermore, a large proportion of IFM is artisanal, and in most cases, the processing lacks the level of sophistication to implement necessary food safety control measures to ensure high level of food safety [7]. Most of the IFM is processed from unpasteurized raw milk, which has been shown to harbor foodborne pathogens [8]. Possible contamination with foodborne pathogens from milk handlers can also occur due to the lack of adequate food safety control measures within the milk processing environment [9,10]. Furthermore, water and sanitation hygiene in most households in developing countries remains a key challenge [11] and may affect the safety of IFM processed at household level.

The movement of milk and milk products along the value chain from farms through intermediate stages such as collection/cooling points to the retail market subjects the products to different hygiene conditions and causes them to undergo several changes including temperature variations [12,13]. On the other hand, changes in pH have been reported to affect the level and group of microorganisms present in IFM [14]. In addition, the fermentation process of IFM from different animals proceeds at different rates, which has the potential to select for different microbiota within the final products [14,15]. Finally, numerous studies have indicated significant differences in IFM microbial quality at different points in the value chain [16,17]. All these factors have the potential to variably affect the microbial safety of IFM.

The mounting evidence showing that production practices and other factors may contribute to contamination and proliferation of pathogens in IFM necessitates the use of a structured and transparent approach to collate the available but fragmented data on the microbiological safety of IFM from various sources. Meta-analysis may provide the means by which data from multiple studies can be combined to derive pooled prevalence estimates for different foodborne pathogens in IFM and to identify causes of heterogeneity among data.

Globally, meta-analyses have largely focused on the effects of fermented milk on the health of consumers including cardiovascular diseases and cancer [18]. Literature reviews of various technologically important microorganisms and probiotics in fermented milk have also been carried out [19,20]. The existing gap regarding microbial safety of IFM undermines other research such as quantification of global and regional burdens of disease and risk factors attributed to foodborne pathogens. It is for these reasons that we carried out a meta-analysis to estimate the prevalence of foodborne pathogens in IFM by utilizing the rich diversity of IFM in Africa as a case. We also evaluated the level and sources of heterogeneity among published data through meta-regression.

## 2. Materials and Methods 

### 2.1. Literature Search

A systematic search strategy was used to identify studies reporting the prevalence of pathogens in IFM in Africa. The search was made in Pubmed, Commonwealth Agricultural Bureaux (CAB) Abstracts, Web of Science, Scopus and African Journals Online (AJOL) databases. The search was carried out in May 2017 using the terms “fermented milk pathogens”, “fermented milk microorganisms”, “microbial quality fermented milk”, and “country name” as the string of keywords for studies between 2000 and 2017. Mendeley Desktop version 1.17.13 was used to catalogue, collate, and manage the collected publications and citations thereafter.

It had previously been stated that differences in developmental and economic status and in level of hygiene can result in heterogeneity in the prevalence of pathogens in foods among African countries [21]. In order to assess the contribution of some of these factors to the prevalence of pathogens in IFM, data related to socioeconomic, water, hygiene, and sanitation status of African countries including gross domestic product from the FAOSTAT database [22] and water, hygiene, and sanitation from the UNICEF database [23] were collected.

### 2.2. Inclusion and Exclusion Criteria

Two authors independently screened the collected publications to identify potential studies, and all authors were involved in collating the extracted information from the publications. Any included article had to be published between 2000 and 2017; to report prevalence of one or more pathogens in IFM from Africa analyzed and confirmed by any technique; and to have the IFM be from any livestock species including, cattle, camel, and goats. Publications were excluded if they were in duplicate; assessed the physiochemical properties of IFM; or only described the isolation, identification, and characterization of fermentative microorganisms in IFM. They were also excluded if they described either the technological properties of fermentative microorganisms isolated from IFM or interventions to reduce pathogens in IFM. Studies that solely assessed the physiochemical and microbial changes due to application of specific starter cultures in IFM, shelf-life studies of IFM, and pathogens in fermented milk products besides IFM were also excluded. Finally, studies in which the results were either pooled together with results of raw milk and other milk products or reported inexplicitly or as concentration instead of prevalence were excluded. 

### 2.3. Data Extraction

Full text articles and abstracts were screened independently, and data were extracted into a pretested spreadsheet. Data of author(s), country of study, pathogen name(s), pathogen prevalence (calculated as a percentage of total cases that were positive), animal species from which the milk came from, media of pathogen pre-enrichment, enrichment and isolation, and method of pathogen confirmation/identification were extracted independently using this spreadsheet. Other meta-data that included pH, temperature, and antibiotic resistance were also extracted. For each of the identified countries, socioeconomic, water, hygiene, and sanitation data were extracted from the World Bank, FAOSTAT, and UNICEF databases.

### 2.4. Data Analysis

Data analysis was carried out in R statistical version 3.2.3. A cluster analysis was carried out using the socioeconomic, water, hygiene, and sanitation data collected from grey literature search. Hierarchical cluster analysis was performed using the Euclidean distance between the predictor variables. Meta-analysis, meta-regression, and the accompanying graphical presentation were carried out with R’s *metafor* and *meta* packages [24,25]. Random-effects meta-analyses were carried out to estimate the prevalence (expressed as a percentage) of pathogens in IFM, which was estimated from the number of cases against the total number of positives. The Hunter–Schmidt τ^2^ estimator was used to estimate the amount of heterogeneity while the Higgins *I*^2^ statistic was used to estimate the percentage of total variability due to heterogeneity. The results were presented as forest plots using the *forest* function. Due to the high degree of heterogeneity between the studies for all subcategories (*I^2^* > 75%), the meta-regression was carried using a random-effects model as opposed to fixed effects model. The need for a random-effects model was tested by the full versus reduced model comparison using a likelihood ratio test via *anova* function of *metafor*. To account for the sources of the heterogeneity, moderators were fitted into the model, which used the restricted maximum-likelihood estimator method. The analysis followed a stepwise backwards and forwards approach based on the lowest Akaike information criterion (AIC). R^2^ value was obtained for the final prediction equation. Funnel plots were plotted to assess the presence of heterogeneity and possible publication bias for a random-effects model with and without moderators. Cumulative meta-analysis was carried out to illustrate how the prevalence of pathogens in IFM changed from 2004 to 2017 using the *cumul* function of *metafor*. The results were presented as a cumulative forest plot.

## 3. Results

### 3.1. Staphylococcus spp., Pathogenic E. coli, L. Monocytogenes, and Salmonella spp. were the most Reported Foodborne Pathogens in IFM

**Figure 1:**
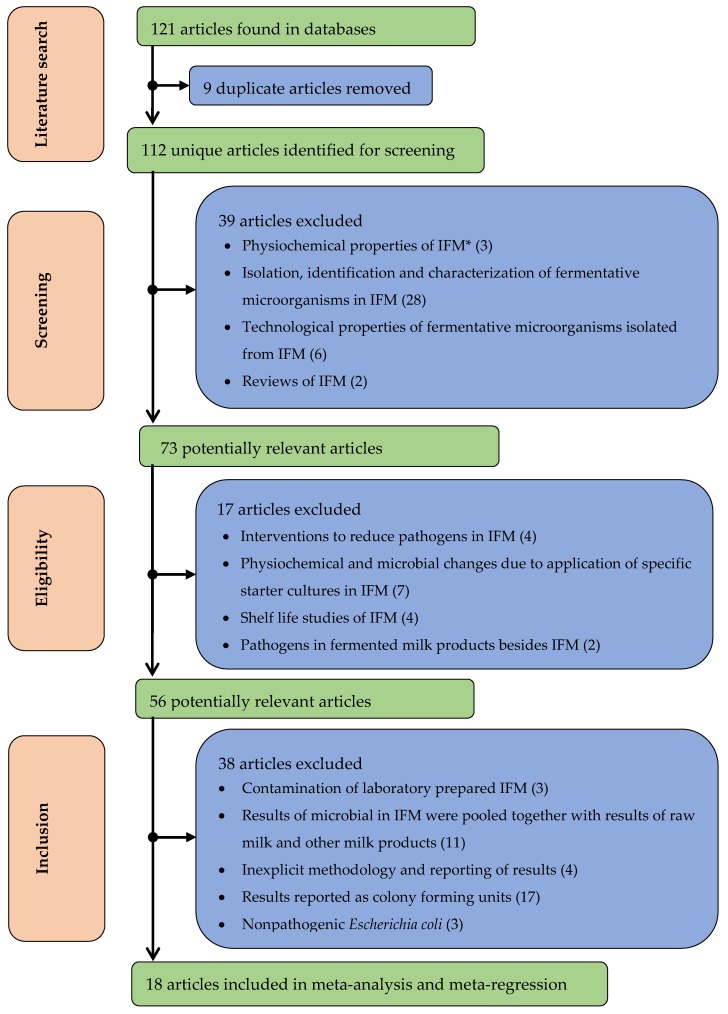
A framework for identification, screening, and inclusion of articles for meta-analysis and meta-regression. * IFM; indigenous fermented milk.

For our analyses, we searched for articles publishing data on foodborne pathogens in IFM. We used Africa as a case study and carried out a systematic literature search, which yielded 121 publications. Out of these, we selected 18 publications from 15 countries through the exclusion–inclusion criteria presented in Figure 1. The pathogens mostly analyzed and reported in the publications included *Staphylococcus* spp., pathogenic *E. coli*, *L. monocytogenes*, and *Salmonella* spp. (Table 1). The pathogenic *E. coli* included Shigatoxin-producing *E. coli* O157:H7, *Shigella* enterotoxin-producing O8:H19, and enterotoxigenic strains.

### 3.2. Staphylococcus aureus and Pathogenic Escherichia coli were the Most Prevalent Pathogens in Published Studies

**Figure 2:**
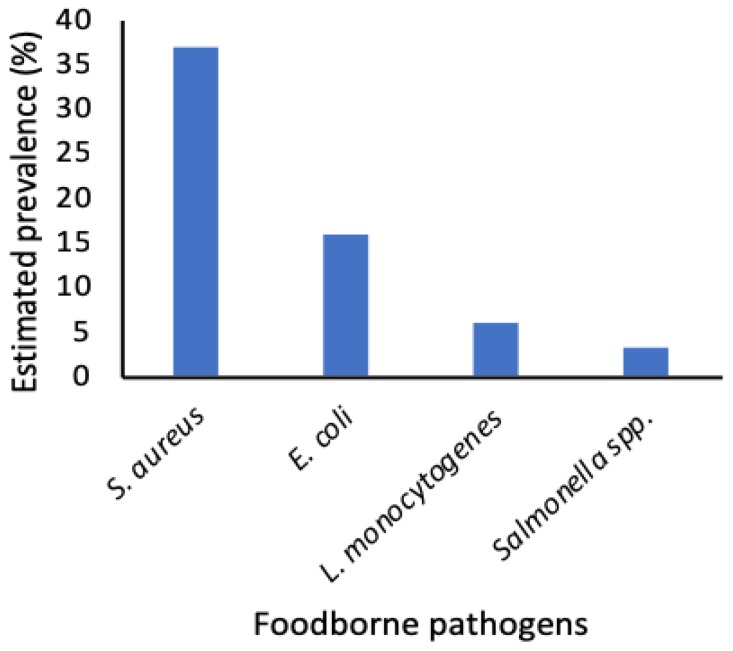
Prevalence estimates of most frequently reported foodborne pathogens in African indigenous fermented milk.

**Figure 3:**
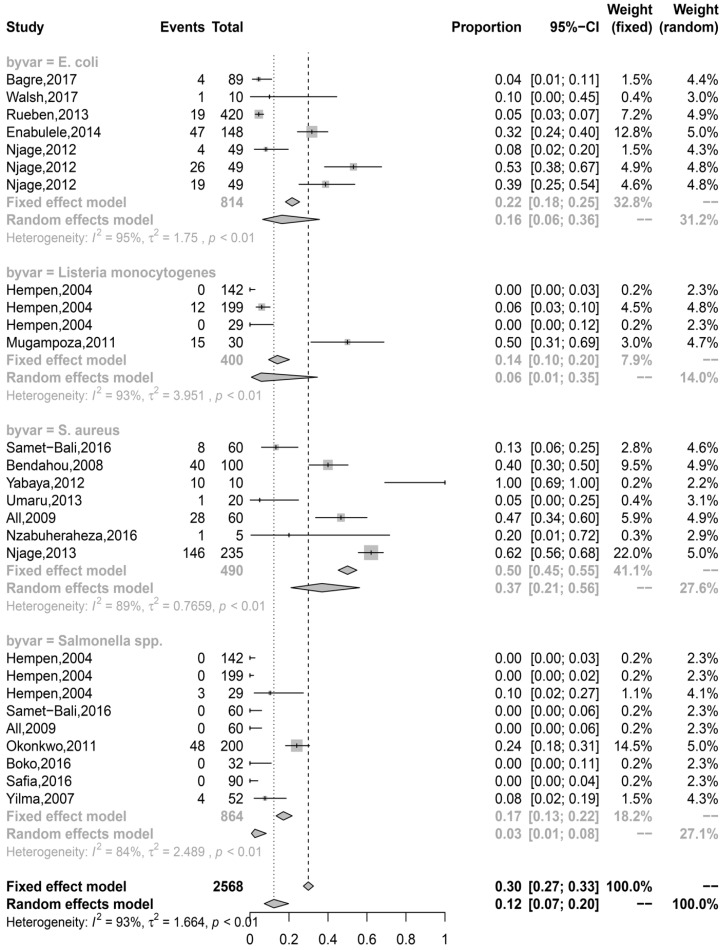
Forest plot of prevalence of pathogens in African indigenous fermented milk: The grey square around the dot represents the contribution of each study (weight) to the meta-analysis, and the center dot represents point estimates. Grey font defines the models, fixed effect and random effects, used to estimate the prevalence of each pathogen group that is denoted as ‘by var= ’.

We then sought to pool the prevalence data from the selected studies in order to estimate their overall prevalence in the IFM. The estimates are summarized in Figure 2. Among the four pathogens, our estimates showed that *S. aureus* was the most prevalent at 37% (range = 5% to 62%) followed by pathogenic *E. coli* at 16% (range = 4% to 53%). The estimates for *L. monocytogenes* and *Salmonella* spp. were 6% (range = 0% (not detected) to 50%) and 3% (range = 0% (not detected) to 24%), respectively. Our overall prevalence estimate for the four foodborne pathogens was 12% (95% confidence interval = 7–20%). Given that the estimates were made from different studies, we sought to determine to what extent the reported prevalence differed among the published studies. In this case and as presented in Figure 3, we found that the prevalence of the pathogens was significantly heterogeneous among different studies (*p* < 0.01).

### 3.3. IFM Metadata in Published Studies 

The interaction between intrinsic and extrinsic factors affect the microbial quality of a food product, while sampling and analytical methods can affect the isolation and identification of microorganisms. The interplay among these factors contributes to the reported microbial prevalence in a food product. For these reasons, we were prompted to explore and describe related meta-data from the 18 published studies as possible factors that could influence the prevalence of foodborne pathogens in the IFM. We sought to identify the animal from which the milk was obtained from, sampling point, method used to confirm pathogens, as well as physiochemical data such as pH and temperature at the time of sampling.

We identified that the sampling was carried out at four points of the IFM value chain. These included cooling points, production, market and retail, and household, with market and retail being the most frequently sampled (Table 2). Apart from two studies that analyzed IFM from camel, all the others analyzed IFM from cow milk. The pathogens were identified by either PCR-sequencing or biochemical tests, the latter being the most utilized. The pH of IFM samples was reported in six studies, and it ranged from 3.3 to 6.0. In one study, the temperature of IFM samples from market places in Gambia, Senegal, and Guinea ranged from 13–34 °C [30]. Our averaged mean calculated from individual means provided in the studies for temperature in the market and pH (Table 2) resulted to 29.8 ± 1.3 °C and 4.2 ± 0.2, respectively. In another study from Uganda, the mean temperature of IFM at the cooling point was 8.6 ± 0.4 °C [31]. The 18 articles thus provided a wide range of data that could be utilized in meta-regression analysis to determine the causes of heterogeneity identified in Figure 3. Despite the hypothesis that various socioeconomic factors, sanitation, water, and hygiene levels may influence the microbial quality of foods in developing nations; only one study provided data on water quality alongside microbial quality of IFM [40]. Hence, evidence for this hypothesis remains elusive. Therefore, we sought to solve this by obtaining data for the 15 countries, which allowed us to cluster the countries into five clusters based on their socioeconomic, sanitation, water, and hygiene data (Supplementary file 1). Cluster 1 comprised of Algeria, Egypt, Morocco, and Tunisia. Cluster 2 comprised of Benin and Ethiopia. Cluster 3 comprised of Ghana, Guinea, Kenya, Nigeria, Senegal, and Uganda. Cluster 4 comprised of Burkina Faso, while cluster 5 comprised of Gambia and Rwanda. Similarly, the clusters could be utilized in meta-regression analysis.

### 3.4. Prevalence Estimates were Highly Associated with the Point of Sampling, Country Clusters, and Pathogens in IFM

Having identified that the overall prevalence estimate of the four pathogens in IFM was heterogeneous (Figure 3), we hypothesized that the available meta-data (Table 2) could account for the heterogeneity. This was based on the premise that varying factors, which can either be biological or methodological, may contribute to the heterogeneity in estimates obtained from meta-analysis [41]. In our case, we used the data presented in Table 2 and country clusters to carry out univariate and multivariate analyses. The univariate analysis revealed that point of sampling, microbial group, and country clusters were significantly associated with heterogeneity in our prevalence estimate (*p* < 0.05) (Table 3). In this regard, IFM sampled at milk collection points had a significantly higher prevalence estimate than at market and retail, household, and production points (*p* < 0.05). Estimates at the household level had the least estimates compared to milk collection points. On the other hand, estimates of pathogenic *E. coli* were significantly lower than *S. aureus* (*p* < 0.05) but statistically similar to those of *L. monocytogenes* and *Salmonella* spp. (*p* > 0.05). Finally, prevalence estimates of country cluster 1 were significantly lower than country clusters 2 and 5 (*p* < 0.05) but similar to country clusters 3 and 4 (*p* > 0.05). Multivariate meta-regression model also showed that point of sampling and microbial groups were significantly associated with our prevalence estimates (*p* < 0.05) in a trend similar to the univariate analysis (Table 3). 

### 3.5. Publication and Heterogeneity Bias

**Figure 4:**
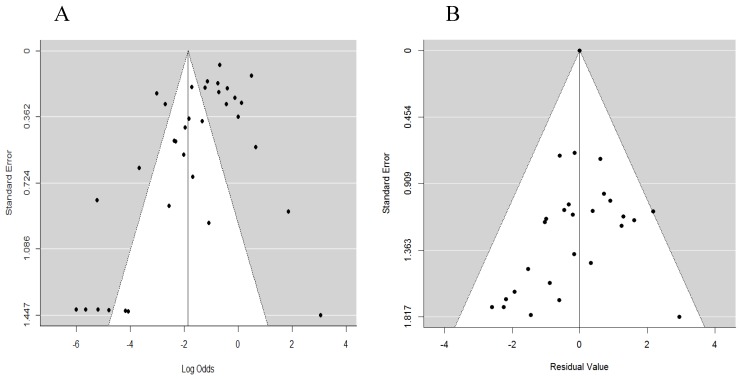
Funnel plots for examination of heterogeneity/publication bias with (**A**) a model without moderators (random-effects model) and (**B**) a model with moderators (mixed-effects model).

It has been previously stated that factors such as selection bias, true heterogeneity, data irregularities, as well as chance can contribute to publication and heterogeneity bias [41]. We, therefore, sought to determine the extent of bias in our study and whether the specific factors identified in Table 3 moderated the bias. In this case, we carried out an estimate without moderators (random-effects model) and another with moderators (mixed-effects model). As demonstrated with funnel plots in Figure 4, we could illustrate the extent of the bias in the published articles that we selected for our analyses. Not only is the funnel in Figure 4A asymmetrical but also a majority of the points fall outside the funnel. By including meta-data in Table 3 as moderators, we were able to obtain a symmetrical funnel (Figure 4B), thus showing that indeed year of publication, country cluster, pathogen group, point of sampling, and method of confirmation were the cause of the bias. The final model, which included moderating variables, accounted for 63% of the variance. The residual heterogeneity was 90%.

### 3.6. The Prevalence of Foodborne Pathogens in IFM was Dynamic over Time

**Figure 5:**
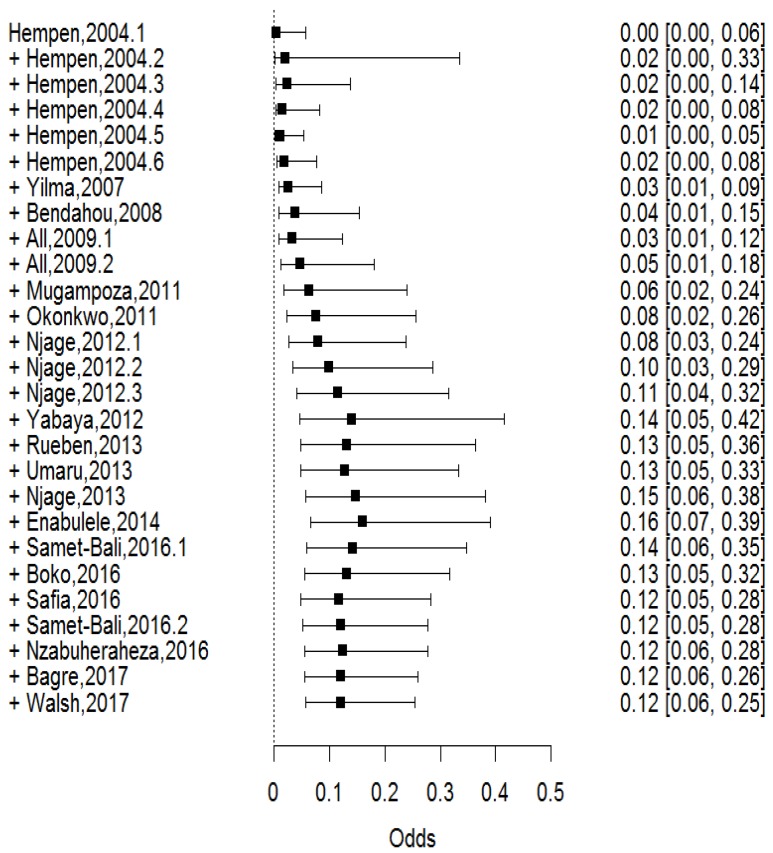
Forest plot showing the results from a cumulative meta-analysis of 18 studies assessing the prevalence of pathogens in African indigenous fermented milk between 2004 and 2017.

Prevalence estimates from multiple studies can reveal trends over time, which in turn can give insight into the underlying causes of a given situation and provide insightful information about recent developments within a thematic area [42]. We, therefore, postulated that the prevalence of foodborne pathogens in IFM was dynamic over our specified period of study, that is, 2000 to 2017. To resolve this, we carried out a cumulative meta-analysis, which determined the change in odds of IFM contamination in a chronological sequence of the selected studies. As seen in the forest plot (Figure 5), we indeed showed that the prevalence of pathogens in IFM increased from 2004 to 2017. In 2017, the odds of contamination of IFM were nine times higher than in 2004. A closer look at the forest plot revealed that the odds rose markedly from 2010 to a peak in 2014 and then declined steadily to 2017. We noted that this corresponded to a higher number of studies between 2010 and 2014 than before 2010 and after 2014. This was an indication that, as research output in IFM increased, the significance of microbiological safety of IFM was unraveled.

### 3.7. An Insight into the Antibiotic Resistance Risk from Pathogens in IFM

Finally, we sought to describe the antibiotic risk that may arise from foodborne pathogens in IFM. This is because pathogens that are resistant to antibiotics, especially the multidrug-resistant pathogens, present major public health and economic concerns worldwide [8]. We identified only three out of the 18 studies that went further to characterize the antibiotic profile of pathogens isolated from IFM. In one study, three *E. coli* strains were resistant to two or more antibiotics, whereby one strain was resistant to tetracycline and sulfonamide while two strains were resistant to amoxicillin, nalidixic acid, and tetracycline [28]. In another study [27], all *E. coli* O157 strains isolated from IFM were multidrug resistant. Notably, 100% were resistant to penicillin and tetracycline; 84.2% were resistant to amoxicillin, oxacillin, and sulphamethoxazole/trimethoprim; and 68.4% were resistant to chloramphenicol and 42.1% to streptomycin. In the third study, 0.3% of the isolates were Methicillin-Resistant *Staphylococcus aureus* (MRSA) [35]

## 4. Discussion

Due to the fragmentation of data related to IFM microbial safety, we set out to determine the pooled prevalence of foodborne pathogens in IFM and to determine factors that could affect the estimates using meta-analysis and meta-regression approaches. We used Africa as a case due to the high consumption and rich diversity of indigenous fermented foods in the region, including IFM [19,20,43,44].

As evident in Figure 1, the microbiological safety of African IFM has been investigated in different studies. However, the number of studies that met our inclusion–exclusion criteria was low. This seems to reflect a general obstacle when conducting meta-analysis in Africa for foodborne pathogens given that a study on different food groups from Africa selected only 66 publications for its metanalysis [21] and that another study that sought to identify *E. coli* 0157 in cattle only included four studies from Africa [41]. Most studies in African institutions of higher learning are unpublished [21], while two-thirds of the published studies are in local journals that do not feature in international databases [45]. These factors may account for the few publications in our present study. Nonetheless, the 18 publications are comprised of 1980 IFM samples taken from different points of the IFM value chain; hence, we could reliably conduct the meta-analysis and meta-regression.

Our study identified *S. aureus*, pathogenic *E. coli*, *L. monocytogenes*, and *Salmonella* spp. as the most reported foodborne pathogens in IFM. In order of prevalence, *S. aureus* > pathogenic *E. coli* > *L. monocytogenes* > *Salmonella* spp. (Figure 1). Presence of *S. aureus* in IFM points to low hygiene and sanitation practices during handling or processing of IFM because *S. aureus* is associated with poor hand hygiene practices by food processors and vendors [21,46] and numerous studies have reported the occurrence of low hygiene practices in African milk value chains [47,48]. The contamination may also occur due to *S. aureus* shedding from clinical or subclinical mastitis udder during milking, which is a huge problem whereby, in some African countries, the prevalence of clinical and subclinical mastitis in lactating livestock is 4 and 90%, respectively [49,50]. On the other hand, *E. coli* is ubiquitous in the intestinal tracts of mammals [51] and, hence, may point to fecal contamination of the IFM. A recent study found that pastoralists in Ethiopia did not follow any sanitary procedure when milking animals and that, often, their hands were soiled with feces from the animals [52]. This further implicates low levels of hygiene and sanitation practices to the high detection of both *S. aureus* and pathogenic *E. coli*.

The detection of *L. monocytogenes* and *Salmonella* spp. in IFM points to exogenous contaminants. One possible cause is contamination of raw milk since farms are particularly known to be reservoirs of both pathogens [53]. Studies from different African countries found that the prevalence of *L. monocytogenes* in raw milk from dairy farms was 2.0–8.8% [54,55] while that of *Salmonella* spp. was as high as 17.7% for cow milk [56] and 43% for camel milk [57]. Raw milk is considered an important vehicle for transmission of *Salmonella* spp. [58]. In addition, some strains of *L. monocytogenes* are known to persist on contact surfaces [59]. In many African countries, IFM is processed, transported, and stored in reusable containers made from locally available materials such as wood fiber, clay pots, and plastic containers [1,60,61,62]. *L. monocytogenes* are known to persist on some of these materials [63], hence contributing to contamination of IFM.

Besides the four pathogens, other important and emerging pathogens, such as *Bacillus cereus*, *Shigella* spp., and *Serratia marcenscens* were reported, and their prevalence was 30.3%, 8.0%, and 4%, respectively [30,31,38]. *Streptococcus* spp. was also reported in IFM [64,65]. However, we excluded these pathogens from the analyses due to lack of sufficient data for meta-analysis and meta-regression. Their exclusion does not necessary signify that they pose a lower risk than *S. aureus*, pathogenic *E. coli*, *L. monocytogenes*, and *Salmonella* spp. but do point to further food safety risks that warrant research attention in future.

From our estimates, we found that the overall prevalence of the four pathogens was 12%. Our findings were lower than those estimated for other foods in Africa [21] but reveal a considerable risk from IFM given that some of the target consumer groups for IFM include children, the old, and the immuno-compromised [66,67,68]. This is particularly important considering that the 95% confidence interval of some estimates were as high as 38–67% and 69–100% for pathogenic *E. coli* and *S. aureus*, respectively (Figure 2). *S. aureus* is a major concern since it is among the leading causes of disease outbreaks related to food consumption and several outbreaks have been linked to its presence in different food products worldwide [69,70,71,72].

Although the other three pathogens were estimated to be lower than *S. aureus* (Table 3), they are also important foodborne pathogens in milk and milk products. Numerous studies have directly linked not only *S. aureus*-related outbreaks but also *E. coli* 0157-, *L. monocytogenes-*, and *Salmonella* spp.-related outbreaks to milk products [73,74,75,76,77,78]. Recent reviews have indicated that *E. coli* 0157 and *Salmonella* spp. are amongst the most important pathogens in Africa [79,80]. On the other hand, it was estimated that the cost of a recent listeriosis outbreak in South Africa was in excess of US $15 million [81]. Although the burden of disease and cost of foodborne outbreaks associated with IFM in Africa remain unknown, contamination with the four major pathogens and other pathogens indicates that IFM may contribute to these public health and economic issues. This is further compounded by the antibiotic resistance data for multidrug-resistant MRSA and *E. coli* 0157 that we identified in three publications

Our study revealed that the sampling point was pivotal in the variation of the pathogen estimates (Table 3). Milk sampled at the collection points showed the highest prevalence estimates. This is based on data available for *L. monocytogenes* in Uganda (prevalence = 50.0%) [31] and *E. coli* 0157:H7 in Kenya (prevalence = 53.1%) [17]. Transportation of IFM to collection points takes many hours and is, in most cases, carried out in ambient temperatures due to lack of refrigeration [82]. Lack of refrigeration may also be attributed to the contamination at the retail or market points. In Kenya, for example, camel IFM is produced in the pastoral areas and sold in Nairobi and its environs, and the distance between the two can be as far as 500 km [47]. A study carried out between the two regions showed that 25% of the milk in pastoral area’s market was unacceptable compared to 75% at the final market in Nairobi [83]. Based on our calculations from Table 2, the temperature of milk at the market place was on average 29.8 °C, which is well within the growth temperature ranges for most of the four pathogens. This is coupled with the long-distance, mimic incubation condition that would be ideal for growth of the pathogens. On the other hand, the low prevalence at the household has been attributed to IFM production practices at the household level whereby utensils used to prepare fermented milk are smoked before fermentation for flavor attributes, which coincidentally produce antimicrobial products [44].

Interestingly, the country clusters which contained data on socioeconomic, water, sanitation and hygiene showed a significant contribution to the heterogeneity in the univariate analysis (Table 3). Clusters 2 and 5 had significantly higher estimates compared to clusters 1, 3, and 4. Therefore, improved sanitation, hygiene, and water quality and high socioeconomic status may correlate with safe foods within a country. This supports the previous untested hypothesis that these factors contribute to the safety of foods in Africa [21] and may help explain some of the observations in the extracted data. For example, *Salmonella* spp. was not detected in Egyptian, Algerian, and Tunisian markets [5,32,37].

The average pH of IFM presented in Table 2 ranged between 3.8–4.6, thus showing that the pH varied widely. This is even more compelling when you consider that data extracted from some publications showed that the pH of some IFM samples was as high 6.4 [30]. All these indicate diversity in the acidification process of IFM and failure in the fermentation process in the IFM samples with high pH whereby the fermented milk does not reach the desired acidification level. Although the pH of IFM is expected to reduce the growth of pathogens [5], some strains of pathogenic *E. coli*, including *E. coli* O157:H7, are able to adapt and develop tolerance to acidic pH levels that would be otherwise lethal [84]. Similarly, some serovars of *Salmonella*, such as *S. Typhimurium*, are adaptable to acidic conditions [85,86]. The same is true for some strains of *L. monocytogenes* [6]. Therefore, failure to achieve the right pH within a short time would considerably increase pathogen risk in IFM.

The two parameters identified in Table 2, pH and temperature, represent key intrinsic and extrinsic parameters that may affect that presence and survival of foodborne pathogens in IFM, particularly during distribution and storage. A combination of low temperature and low pH can be used as a hurdle technology to enhance the safety of IFM. Nonetheless, adaptation to sublethal or mild stresses may enhance the survival of pathogens in lethal stresses as was demonstrated in the case of *L. monocytogenes*, whereby short-term exposure to pH of 5.5 enhanced the survival in pH of 3.5 [87]. The ability of foodborne pathogens to adapt to various stresses [86,87,88] may explain the results in some of the studies whereby, despite the pH and temperature being 4.4 and 8.6 °C, respectively, the prevalence of *L. monocytogenes* was 50% [31] while *S. aureus* and *Salmonella* spp. were detected in pH that was less than 4.0 [5,34,36].

The odds of contamination were highest after 2010 than before 2010 (Figure 5). This may not necessarily indicate that the safety of IFM has reduced over the years, but it shows that, as more data becomes available, the significance of microbiological safety of IFM is unraveled. In this regard, a higher number of articles were published between 2010 and 2017 than between 2000 and 2009 (Table 1). Furthermore, by reviewing the analytical methods used in the studies that we included in our analyses, we identified that, in early 2000s, biochemical tests were mostly used to characterize and confirm pathogens but that there was considerable shift to molecular techniques from 2010 (Table 2). This clearly indicates that advanced analytical methods as well as research output can play a critical role in uncovering new insights into the microbiological safety of IFM.

We have estimated the prevalence of foodborne pathogens in the present study, but there were limitations; hence, present results must be interpreted with caution. First, some variables were reported in a few studies. For example, household and collection points were reported in one and two studies, respectively. Secondly, only one study was found per country in most cases despite there being different IFMs in one country. For example, in Kenya, there are four documented IFMs including *mursik*, *kule naoto*, *amabere amaruranu*, and *suusac* [44], but we only obtained data for *suusac* [16,17]. Furthermore, the selected studies were only limited to those archived in international databases. In addition, our estimates were based only on four of the most reported pathogens. It is particularly important to note the wide data ranges for the pathogens even in similar sampling points within the same country, for example, in Nigeria, the prevalence of *E. coli* O157:H7 in market places was independently reported as 4.5% and 31.8% [24,25]. For these reasons, our study does not give an optimal reflection of foodborne pathogens in African IFM but provides the much-needed data-based baseline for future studies.

## 5. Conclusions

IFM is a diverse and important nutrition source across the African continent, but an increasing number of reports on microbiological data on IFM has raised concern about its microbial safety. This has necessisted the need for unified evidence from the very highly fragmented research characterized by individual and often small studies. Therefore, our study sought to estimate the prevalence of foodborne pathogens in IFM from Africa based on meta-analysis and meta-regression of published data. Out of 121 studies published between 2000 and 2017, only 18 met our inclusion and exclusion criteria. From the studies, we identified *S. aureus*, pathogenic *E. coli*, *L. monocytogenes*, and *Salmonella* spp. as the most prevalent pathogens in IFM. Point of sampling and microbial group emerged as good predictors of the overall prevalence of pathogens in IFM. We also identified that IFM in collection points had higher prevalence estimates compared to household, production, and retail and market points. In summary, we have provided an insight into the microbial safety of African IFM value chain and provided data that might be used in quantitative microbial risk assessment models, development, and implementation of hygiene and safety practices in hot spots along the IFM value chain and other data-driven research. Potential areas that warrant research include microbial safety of diverse IFM in regards to production practices, sampling points, and type of milk used for fermentation, among others. In addition, few studies have screened for antibiotic resistance profile and genetic determinants in pathogens isolated from IFM; hence, more data is needed.

## Figures and Tables

**Table 1:** List of 18 studies selected for meta-analysis and meta-regression indicating the prevalence of the four mostly published foodborne pathogens in African indigenous fermented milk.

Pathogen	IFM Samples	Positive Samples	Prevalence (%)	Country	Reference
Pathogenic E. coli*	148	47	31.8	Nigeria	[26]
Pathogenic *E. coli*	49	4	8.2	Kenya	[17]
Pathogenic *E. coli*	49	26	53.1	Kenya	[17]
Pathogenic *E. coli*	49	19	38.8	Kenya	[17]
Pathogenic *E. coli*	420	19	4.6	Nigeria	[27]
Pathogenic *E. coli*	89	4	4.5	Burkina Faso	[28]
Pathogenic *E. coli*	10	1	10.0	Ghana	[29]
*L. monocytogenes*	142	0	0.0	Gambia	[30]
*L. monocytogenes*	199	12	6.0	Guinea	[30]
*L. monocytogenes*	29	0	0.0	Senegal	[30]
*L. monocytogenes*	30	15	50.0	Uganda	[31]
*S. aureus*	60	28	46.7	Egypt	[5]
*S. aureus*	100	40	40.0	Morocco	[32]
*S. aureus*	235	146	62.1	Kenya	[16]
*S. aureus*	5	1	20.0	Rwanda	[33]
*S. aureus*	60	8	13.3	Tunisia	[34]
*S. aureus*	20	1	5.0	Nigeria	[35]
*S. aureus*	10	10	100.0	Nigeria	[36]
*Salmonella* spp.	60	0	0.0	Egypt	[5]
*Salmonella* spp.	32	0	0.0	Benin	[37]
*Salmonella* spp.	142	0	0.0	Gambia	[30]
*Salmonella* spp.	199	0	0.0	Guinea	[30]
*Salmonella* spp.	29	3	10.3	Senegal	[30]
*Salmonella* spp.	200	48	24.0	Nigeria	[38]
*Salmonella* spp.	90	0	0.0	Algeria	[39]
*Salmonella* spp.	60	0	0.0	Tunisia	[34]
*Salmonella* spp.	52	4	7.7	Ethiopia	[40]

* Pathogenic *E. coli* included Shigatoxin-producing *E. coli* O157:H7, enterotoxigenic *E. coli*, and *Shigella* enterotoxin 2-producing *E. coli.*

**Table 2:** Meta-data of indigenous fermented milk derived from published articles.

Pathogen	Sampling Point	Microbial Confirmation	Mean pH	Mean Temperature (°C)	Reference
Pathogenic E. coli*	Market and retail	PCR			[26]
Pathogenic *E. coli*	Market and retail				[28]
Pathogenic *E. coli*	Production	PCR			[17]
Pathogenic *E. coli*	Collection	PCR			[17]
Pathogenic *E. coli*	Market and retail	PCR			[17]
Pathogenic *E. coli*	Market and retail	Biochemically			[27]
Pathogenic *E. coli*	Production	Biochemically			[29]
*L. monocytogenes*	Market and retail	Biochemically	4.2	28.6	[30]
*L. monocytogenes*	Market and retail	Biochemically	4.1	29.2	[30]
*L. monocytogenes*	Market and retail	Biochemically	4.6	31.7	[30]
*L. monocytogenes*	Collection	Biochemically	4.4	8.6	[31]
*S. aureus*	Market and retail	Biochemically	3.9		[5]
*S. aureus*	Market and retail	PCR			[32]
*S. aureus*	Market and retail	PCR			[16]
*S. aureus*	Market and retail	Biochemically			[33]
*S. aureus*	Market and retail	Biochemically	3.9		[34]
*S. aureus*	Household	Biochemically			[35]
*S. aureus*	Market and retail	Biochemically	3.9		[36]
*Salmonella* spp.	Market and retail	Biochemically			[5]
*Salmonella* spp.	Production	Biochemically	3.8		[37]
*Salmonella* spp.	Market and retail	Biochemically			[30]
*Salmonella* spp.	Market and retail	Biochemically			[30]
*Salmonella* spp.	Market and retail	Biochemically			[30]
*Salmonella* spp.	Market and retail	Biochemically	4.3		[38]
*Salmonella* spp.	Market and retail	Biochemically			[39]
*Salmonella* spp.	Market and retail	Biochemically	3.9		[34]
*Salmonella* spp.	Production	Biochemically			[40]

* Pathogenic *E. coli* included Shigatoxin-producing *E. coli* O157:H7, enterotoxigenic *E. coli*, and *Shigella* enterotoxin 2-producing *E. coli.*

**Table 3:** Meta-regression for the prevalence of pathogens in African indigenous fermented milk.

	Univariate Analysis					Multivariate Analysis				
Variables and Covariates	Estimated Prev Dif	SE	95% CI (LB)	95% CI (UB)	*p*	Estimated Prev Dif	SE	95% CI (LB)	95% CI (UB)	*p*
Intercept	−69.86	193.90	−449.88	310.17	0.72	−26.10	190.28	−399.04	346.85	0.89
Confirmation method										
Biochemical tests (ref)	-	-	-	-	-	-	-	-	-	-
PCR	−0.05	0.79	−1.59	1.50	0.95					
Point of sampling										
Collection point (ref)	-	-	-	-	-	-	-	-	-	-
Household	**−6.56**	**2.03**	**−10.55**	**−2.57**	**0.00**	**−6.11**	**1.29**	**−8.51**	**−3.46**	**0.00**
Market and retail	**−2.81**	**1.325**	**−5.42**	**−0.205**	**0.04**	**−3.735**	**1.19**	**−5.88**	**−1.21**	**0.01**
Production	**−2.94**	**1.31**	**−5.52**	**−0.37**	**0.03**	**−2.80**	**1.13**	**−4.89**	**−0.45**	**0.02**
Pathogen										
*E. coli* (ref)	-	-	-	-	-	-	-	-	-	-
*L. monocytogenes*	−0.95	1.22	−3.35	1.44	0.44	−1.25	1.15	−3.51	1.00	0.28
*S. aureus*	**3.01**	**0.95**	**1.15**	**4.87**	**0.00**	**2.23**	**0.79**	**0.68**	**3.78**	**0.00**
*Salmonella* spp.	−0.80	1.01	−2.77	1.18	0.43	−1.18	0.86	−2.86	0.50	0.17
Cluster										
Cluster 1 (ref)	-	-	-	-	-	-	-	-	-	-
Cluster 2	**2.09**	**1.74**	**−1.32**	**5.51**	**0.03**					
Cluster 3	0.21	1.70	−3.12	3.54	0.90					
Cluster 4	−1.08	1.23	−3.49	1.33	0.38					
Cluster 5	**1.86**	**0.91**	**0.07**	**3.65**	**0.04**					
Year	0.03	0.10	−0.15	0.22	0.72	0.01	0.09	−0.17	0.20	0.89

Prev: prevalence; dif: difference; CI: confidence interval; LB: lower bound; UB: upper bound. Bold figures indicate covariates associated at a *p* value < 0.05.

## Supplementary Materials

The following are available online at https://www.mdpi.com/2076-2607/7/11/563/s1, Table S1: Socio-economic, water, hygiene and sanitation data extracted from the World Bank, FAOSTAT, and UNICEF databases for 15 countires included in the present analyses.
